# Nothing Uncommon

**Published:** 1885-08

**Authors:** 


### NOTHING UNCOMMON.

AN UNPLEASANT ACCOMPLISHMENT.—Under this caption, Francis H. Atkins, S. B., [?] M. D., Los Vegas, Mexico, writes to the *New York Record* of a clothing drummer, who is traveling in that section taking measures for suits, and who has an electrical or some other influence about him which, according to the correspondent's own words, "make many of his customers sick during the process of measuring. The young man boasts of having "floored" fully one hundred persons in the last two years."

That sort of thing is quite common about here, and most of this class of persons possess the faculty. The power lies in the tongue, and is the expression of what is commonly called "cheek" or "gall," and the effect, of which the writer speaks, is hereabout, called "being bored." We have known some instances where violent nausea has been excited at the first visit of the "young man," and at a second or third, on the exhibition of the " cheek," brought out strong, it has been known, in some instances, to have excited violent reflex action of the muscles of the lower extremities, with forcible effect on the drummer's posterior aspect, accompanied by a precipitate and undignified exit from the front door. One case is recorded, indeed, of fatal results, to the customer, the "young man" attacked him in an unguarded moment, and when escape was impossible, and talked him to death, and himself hoarse. Fact!

